# ALS Awareness Month — May 2014

**Published:** 2014-05-02

May is Amyotrophic Lateral Sclerosis (ALS) Awareness Month. ALS, also known as Lou Gehrig’s disease, is a progressive, fatal, neurodegenerative disorder of the upper and lower motor neurons. The etiology of ALS is not well understood, and currently there is no cure. Persons with ALS usually die within 2–5 years of diagnosis.

In October 2010, the Agency for Toxic Substances and Disease Registry (ATSDR) launched the National ALS Registry (http://www.cdc.gov/als) to collect and analyze data regarding persons with ALS in the United States. The goals are to determine the incidence and prevalence of ALS, characterize the demographics of those living with ALS, and examine the potential risk factors for the disease. The registry uses data from existing national databases, including the Centers for Medicare and Medicaid Services and the U.S. Department of Veterans Affairs, as well as information provided by persons with ALS through the secure online web portal. Registrants can also take brief online surveys regarding potential risk factors for the disease.

ATSDR is collaborating with the ALS Association (http://www.alsa.org), Muscular Dystrophy Association (http://www.als-mda.org), Les Turner Foundation (http://www.lesturnerals.org), and other organizations to make all persons with ALS and their families aware of the opportunity to register in the National ALS Registry. Additional features have been added to enhance the registry for patients and researchers, including state and metropolitan area–based ALS surveillance to assist in evaluating the completeness of the registry, a research notification system to inform persons with ALS about new research studies, a biorepository study to evaluate the feasibility of collecting biospecimens from enrollees, and mobile apps to help find the nearest ALS clinics and support groups.

